# Nutritional status and associated factors among children with congenital heart disease in selected governmental hospitals and cardiac center,Addis Ababa Ethiopia

**DOI:** 10.1186/s12887-021-03023-1

**Published:** 2021-12-02

**Authors:** Rediet Woldesenbet, Rajalakshmi Murugan, Feven Mulugeta, Tamirat Moges

**Affiliations:** 1 Department of Nursing and Midwifery, College of Health Sciences, Woldia University, Woldia, Ethiopia; 2 School of Nursing and Midwifery, College of Health Sciences, Addis Ababa University, Addis Ababa, Ethiopia; 3 School of Medicine, College of Health Sciences, Addis Ababa University, Addis Ababa, Ethiopia

**Keywords:** Anthropometry, Children, Congenital heart disease, Nutritional status

## Abstract

**Background:**

Children with congenital heart disease are at risk for poor growth and under-nutrition compared with healthy children. The aim of this study was to assess the nutritional status of children with congenital heart disease and associated factors in selected governmental hospitals and cardiac center Addis Ababa, Ethiopia.

**Method:**

Institutional based cross sectional study among 373 children aged under15 years was conducted from February to March; 2021G.c. Data was collected using structured questionnaire and chart review. Z-scores based on WHO reference ranges were used. Anthropometric z-scores based on WHO 2007 reference ranges were generated for each child. Weight-for-age z-scores for children 0–10 years and height-for-age and BMI-for-age z-scores for all children. Binary logistic regression was used for associated factors.

**Result:**

A total of 373 children were participated in this study. The prevalence of wasting and stunting was 144(38.6%) and 134(35.9%) respectively. The prevalence of underweight and malnutrition in children under 10 years was 143(43.1%). Most of the children were diagnosed with VSD (36.7%). Children age group of 13 months-5 years were associated with wasting and underweight [AOR = 0.434, 95%CI: (0.231, 0.816)] and [AOR = 0.360, 95%CI: (0.183, 0.711)] respectively. Children diagnosed with PAH were 1.885 times more likely to be underweight [AOR = 1.885, 95%CI: (1.094, 3.246)]. When the hemoglobin level increases by every unit per g/dl the chance to be wasting and underweight decreases by 13.1 and 18.6%[AOR = 0.869, 95%CI: (0.792, 0.955)] and [AOR = 0.869, 95%CI: (0.792, 0.955)] respectively. The level of SPO2 is associated with stunting and underweight [AOR = 0.970, 95%CI: (0.943, 0.998)] and [AOR = 0.970, 95%CI: (0.943, 0.998)] respectively.

**Conclusion:**

The prevalence of malnutrition in children with CHD is pretty high. Decreased level of hemoglobin and SPO_2_ was found to be associated factors for malnutrition in this case. There need to be a new strategy about including different health professional while care giving.

## Introduction

Congenital heart disease is a congenital abnormality that affects the structure of the heart walls and vessels [1]. Every year, around 1.35 million live babies are diagnosed with CHD around the world [2]. Malnutrition has a significant impact on the likelihood of infection in children with congenital heart disease, which leads to poor treatment outcomes. Preoperative nutritional status has an impact on postoperative results, as well as poor development and a high mortality rate in this group. Nowadays, management is advancing and moving beyond surgery, with medicine focusing on growth, development, nutrition, and improving the quality of life of children with congenital heart disease [3, 4]. Nutrition is crucial in the treatment of chronic illness. It’s one of the most important factors affecting patients’ recovery after surgery and lowering the risk of infection in children with congenital heart disease [5–7].

High energy needs, insufficient energy intake, poor mesenteric perfusion, delayed enteral feeding, and delayed feeding milestones are all variables that raise the risk of malnutrition in children with CHD. Some of the presumptions indicated in studies conducted to elucidate the reason of malnutrition include not knowing their nutritional desires and preferences [8, 9]. Furthermore, children with congenital heart defects have higher resting energy expenditure, particularly prior to corrective surgery [10].

The nutritional assessment results of healthy children and children with congenital heart disease show a significant difference. Stunting and underweight were found to be 58.72 and 82.53% in children with congenital heart disease, respectively, according to an Indian study [1]. Malnutrition was shown to be prevalent in 90.4% of children with CHD and 21.1% of healthy children in a Nigerian study [11]. In a research conducted in Hawassa, Ethiopia, more than half of the children identified with congenital heart disease are malnourished [12].

Despite the fact that there have been numerous studies on malnutrition in Ethiopian children, there are few studies on children with congenital heart disease and their nutritional assessment in particular [12]. The goal of this research is to discover more about the influence of CHD on children’s nutritional status.

The study will make a significant contribution to the development of malnutrition prevention strategies for children with CHD. The purpose of this study is to identify the nutritional status and prevalence of malnutrition in these categories of cases, which will aid policymakers in determining how best to allocate resources to the affected population. The results of this study will aid both government and non-government organizations responsible for child health in terms of planning early intervention based on gaps. The study’s findings will also be useful as baseline data for future researchers.

The aim of this study was assess the nutritional status of children with congenital heart disease and associated factors in selected governmental hospitals and cardiac center Addis Ababa, Ethiopia 2021.

## Methods

### Study setting and population

Institutional based cross sectional study design was conducted in three governmental hospitals Black Lion Hospital, St. Peter specialized Hospital yekatit 12 Hospital and cardiac center of Ethiopia. The study was conducted from February to March, 2021G.c. The study included 395 children under the age of 15 who had been diagnosed with proven congenital heart disease. The number of children counted 3 months before the data collection was 976, and the proportional allocation was done based on this number.

Children diagnosed with congenital heart disease whose age is less than 15 years during the study period and come to cardiac OPD for their follow up was included for the study and children presented with another congenital anomaly related to feeding and children who were critically ill were excluded.

Children: For this study age group is taken as child who aged under 15.

Minimum acceptable dietary diversity was when child have 4 food groups dietary diversity [13].

Based on the WHO standard recommendation a cut-off Z score less than -2SD was taken as malnutrition weight –for-Height Z-score (wasting), weight –for-age Z-score (underweight) and height-for-age Z-score (stunting) [14].

Congenital heart defect: major or minor congenital anomalies defined as anatomical structural and functional defect present at birth which was confirmed by pediatricians with echocardiography.

### Design and data collection

The sample size was calculated using single proportion formula. It is determined by using the prevalence 63% wasted and 29.8% stunted children with congenital heart disease according to the study done in Hawassa, Ethiopia [12]. Based on this assumption, the actual sample size for the study is determined using the formula for single population proportion.

After adding non response rate of 10% of non-response rate the final sample size is approximately 395.

Three of the hospitals (TikurAnbessa Hospital, St. Peter specialized Hospital, Yekatit 12 Hospital) and cardiac center of Ethiopia was selected for conducting the research intentionally. These hospitals were selected because they have cardiac follow up clinic and a better patient load as they are referral hospitals. And also cardiac center is the only center for children with heart disease in Ethiopia also it is the only place they get corrective surgery for CHD. Children come to cardiac follow up to the selected areas 3 months prior to the data collection was counted and the proportional allocation was done based on that. Study subjects were selected by consecutive sampling technique.

Before conducting data collection structured questionnaire was prepared after reviewing the literatures. Pretest was done on 5% of the study subjects at a different Hospital who were not included for the final data the tool was modified accordingly. The data was collected by face to face interview for the socio demographic and dietary history data. Also 24 h recall method was used for detailed history of dietary questions, while child medical history was gathered from medical charts of the child and anthropometric data were measured at the time of data collection.

Data was collected by health professionals (BSc Nurses) who obtained training for the data collection and supervised by the principal investigator and another Nurse. All of them were not working in the institutions where the data was collected.

Anthropometric measurement was assessed as follows

- Infants and children under24 months of age had their lengths measured lying down (supine). Heights of children over 24 months of age was measured while standing to the nearest 0.1 cm. Patient positioning was made with the shoulder blades, buttocks, and heels on the vertical backboard.
- Weight was measured in kilograms. Infants or children who were unable to stand alone on the scale, was measured first as an adult stand on the scale and zero the scale with the adult standing on the scale. Then the child was handed to the adult to obtain an accurate measurement of the child. Children who can stand on the weight scale by themselves were measured with light clothing. Weight was measured to the nearest 0.1 kg

### Data processing and analysis

After editing and sorting the questionnaire’s it was entered to EPI data version 4.6 and analyzed through SPSS version 25.The descriptive data was presented in tables, charts and texts. Binary logistic analysis was done to review the factors associated with nutritional status of children with CHD. Variables with< 0.25 *p*-value on binary was taken to multivariable analysis and variables with < 0.05 *p*- values was considered statistically significant in multivariable analysis. The anthropometric data was entered and analyzed using WHO AnthroPlus tool whose age is 0 to19 years of age.

## Results

### Demographic characteristics of participants

Hundred and fifty-eight (42.3%) of 373 children were aged 6–15 years and 190(50.9%) were female. Of those 373 children, 298(79.9%) had acyanotic heart disease only 51(13.7%) had corrective surgery. Hundred and thirty-eight children were having pulmonary hypertension. (Table 1).

**Table 1:** Socio demographic characteristics of children with congenital heart disease in selected governmental hospitals and cardiac center Addis Ababa, Ethiopia 2021

Variables	Category	Frequency (*n* = 373)	Percent
Gender	Male	183	49.1%
	Female	190	50.9%
Age	0–12 months	76	20.4%
	13 months-5 years	139	37.3%
	6-15 years	158	42.3%
Weight range	–	2.5-45 kg	
Height range	–	49-172.0cm	
Relationship with the child	Mother	255	68.3%
	Father	92	24.7%
	Other	26	7.0%
Residency	Urban	342	91.7%
	Rural	31	8.3%
	Total	373	100%
Family size	< 4	309	82.8%
	> = 4	64	17.2%
Income	<=5000 ETB	227	60.9%
	> = 5001ETB	146	39.1%
Birth order	First order	139	37.3%
	2nd - 3rd	171	45.8%
	4th and more	63	16.9%
Birth interval	> = 24 months	214	90.7%
	< 24 months	22	9.3%

### Dietary information

Among the 132 children that were breastfeeding, 83 (62.9%) of them did so within the first hour of delivery. Ninety-eight (74.2%) are currently nursing, while 96(72%) have begun complementary feeding among this 82 (85.4%) start complementary feeding on 6 months and above 88 (66%) utilize bottle feeding. (Table 2).

**Table 2:** Information about 0–24 months children with congenital heart disease in selected governmental hospitals and cardiac center Addis Ababa, Ethiopia 2021 (*n* = 132

Variable	Categories	Frequency (*n* = 132)	Percentage
**Time when the child was first breastfeed**	Immediately	83	62.9%
	Within first day	21	15.9%
	Within 3 days	9	6.8%
	More than 3 days	19	14.4%
**Colostrum**	Yes	101	76.5%
	No	31	23.5%
**Frequency of breast feeding**	> = 8 times	86	87.8%
	< 8 times	12	12.2%
**bottle feeding**	Yes	88	66.7%
	No	44	33.3%
**Introduction of complementary feeding**	> = 6 months	82	85.4%
	< 6 months	14	14.6%

Approximately 68% of youngsters receive the necessary minimum dietary diversification which is 4 and above food groups from the total.

### Child medical condition

In the preceding 2 weeks, 130 (34.9%) of the participants had been sick. A total of 138 (37%) of the 373 individuals have pulmonary hypertension, and 26 (7%) have heart failure. Corrective surgery was performed on 51 (13.7%) of the children who took part in this study. The average age at which corrective surgery performed was 50.8 months. There were 129 (34.6%) children with acyanotic CHD who also had pulmonary hypertension, and 9 (2.4%) children with cyanotic CHD who also had pulmonary hypertension. (Table 3).

**Table 3:** Medical status of children with congenital heart disease in selected governmental hospitals and cardiac center Addis Ababa, Ethiopia 2021

Medical condition	Categories	Frequency	Percentage
Type of CHD	Acyanotic CHD	298	79.9%
	Cyanotic CHD	44	11.8%
	Acyanotic plus cyanotic CHD	31	8.3%
Sickness in the last two weeks	Vomiting	30	23%
	Diarrhea	30	23%
	Cough/common cold	54	41.5%
	Other	49	37.6%
Pulmonary hypertension	Yes	138	37%
	No	235	63%
Heart failure	Yes	26	7%
	No	347	93%
Corrective surgery	Yes	51	13.7
	No	322	86.3
Acyanotic CHD*PAH^2^	–	129	34.6%
Cyanotic CHD *PAH^1^	–	9	2.4%

### Types of congenital heart disease

**Fig. 1:**
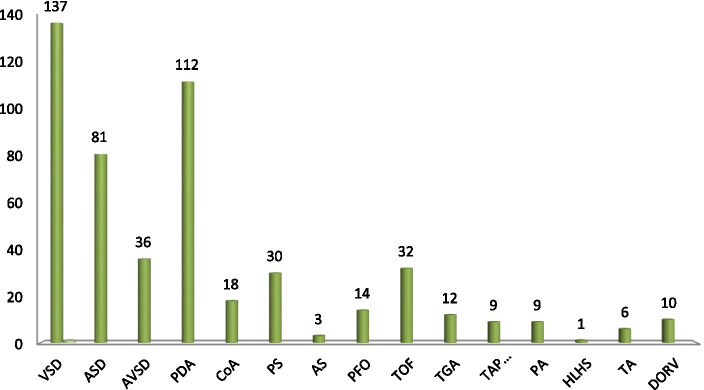
Types of specific congenital heart disease

Among 373 study participants, 298 (79.9%) have acyanotic CHD and 44(11.8%) of them have cyanotic CHD, and the rest 31(8.3%) children diagnosed with both cyanotic and acyanotic CHD at the same time. (see Table 3) Among children with acyanotic congenital heart disease VSD takes the biggest portion which was 137(36.7 %) followed by PDA 112(30%). And from the cyanotic CHD group, TOF 32(40%) is the major one followed by TGA 12(15%). (Fig. 1).

### Nutritional status

The prevalence of wasting and stunting was 144(38.6%) 95%CI (33.64, 43.57%) and 134 (35.9%) 95%CI (31.03, 40.82%) respectively. Underweight was measured for children aged under 10 years and the prevalence was 143(43.1%) 95%CI (37.7, 48.4%). The prevalence of obesity and overweight in this study was 16(4.3%) 95%CI (2.2, 6.3%), and 15(4%) respectively. All three types of undernutrition were predominant among children in the age group of 0-12 months in which the prevalence of underweight, stunting, and wasting was 46(60.5%), 31(40.8%), and 40(52.6%) respectively. Underweight was predominant in children with acyanotic CHD 111(42%) and children with cyanotic CHD stunting was more dominant 18(40.9%). (Table 4).

**Table 4:** Nutritional status of children with congenital heart disease in selected governmental hospitals and cardiac center Addis Ababa, Ethiopia 2021

Variables	Underweight (WAZ < -2) (*n* = 332)		Wasting (BAZ/WFH < -2) (*n* = 373)		Stunting (HAZ < -2) (*n* = 373)	
Sex	Yes	No	Yes	No	Yes	No
Male	74 (45.7%)	88 (54.3%)	80 (43.7%)	103 (56.3%)	67 (36.6%)	116 (63.4%)
Female	69 (40.6%)	101 (59.4%)	64 (33.7%)	126 (66.3%)	67 (35.3%)	123 (64.7%)
**Age**						
0–12 months	46 (60.5%)	30 (39.5%)	40 (52.6%)	36 (47.4%)	31 (40.8%)	45 (59.2%)
13 months-5 years	56 (46.3%)	83 (59.7%)	45 (32.4%)	94 (67.6%)	50 (36%)	89 (64%)
6–15 years	41 (35%)	76 (65%)	59 (37.3%)	99 (62.7%)	53 (39.6%)	105 (66.5%)
**Type of CHD**						
Acyanotic CHD	111 (42%)	153 (58%)	117 (39.3%)	181 (60.7%)	105 (35.2%)	193 (64.8%)
Cyanotic CHD	15 (38.5%)	24 (61.5%)	12 (27.3%)	32 (72.7%)	18 (40.9%)	26 (59.1%)
Acyanotic + cyanotic	17 (58.6%)	12 (41.4%)	16 (51.6%)	15 (48.4%)	11 (35.5%)	20 (64.5%)
Cyanotic CHD * PAH(^a^)	4 (50%)	4 (50%)	3 (33.3%)	6 (66.7%)	5 (55.6%)	4 (44.4%)
Acyanotic CHD* PAH(^b^)	63 (53.4%)	55 (46.6%)	59 (45.7%)	70 (54.3%)	55 (42.6%)	74 (57.4%)

^a^cyanotic congenital heart disease with pulmonary hypertension

^b^acyanotic congenital heart disease with pulmonary hypertension

### Factors associated with nutritional status

Compared to children age 0–12 months, children in the age group 13 months-5 years [AOR = 0.434, 95%CI: (0.231, 0.816)] were 56.6% times less likely to be wasted, and when hemoglobin level increases by every unit in gram per deciliter the chance of being wasted decreases by 13.1% with an adjusted odds ratio [AOR = 0.869, 95%CI: (0.792, 0.955)]. Children who were sick in the previous 2 weeks before the study period were 2.203 times more likely to be wasted [AOR = 2.203, 95%CI: (1.367, 3.551)]. The chance of being wasted increases 1.072 times when the age to getting corrective surgery increases by 1 year [AOR = 0.1.072, 95%CI: (1.001, 1.049)].

In comparison to children whose fathers were employed to governmental or non-governmental jobs, children who had fathers that were merchants were 61.6%less likely to be stunted [AOR = 0.384, 95%CI: (0.157, 0.944)]. The delay in age of the children when getting surgery increases the chance of being stunted by 1.040 times [AOR = 1.040, 95%CI: (1.007, 1.074)]. The odds of being stunted decreases by 3% when the level of SPO2 increases by 1%[AOR = 0.970, 95%CI: (0.943, 0.998)]. Children who were not bottle feed were 2.993 times more likely to be stunted [AOR = 2.993, 95%CI: (1.111, 8.065)].

The chance of being underweight decreases by 18.6% when the level of hemoglobin increases by 1 g/dl [AOR = 0.814, 95%CI: (0.731, 0.906)]. Compared to children who were not sick children who had sickness in the past 2 weeks were 1.834 times more likely to be underweight [AOR = 1.834, 95%CI: (1.043, 3.226)]. The level of SPO2 increases by 1% the chance of being underweight decreases by 6.4%[AOR = 0.936, 95%CI: (0.906, 0.968)]. The odds of being underweight decreased 64% in children age 13 months-5 years compared to children age 0–12 months and also it’s decreased by 56.1% in children 6-10 years[AOR = 0.360, 95%CI: (0.183, 0.711)] and [AOR = 0.439, 95%CI: (0.212, 0.909)] respectively. Also, children diagnosed with pulmonary hypertension were 1.885 times more likely to be underweight [AOR = 1.885, 95%CI: (1.094, 3246)]. (Table 5).

**Table 5:** Binary logistic regression and multivariable analysis among children with congenital heart disease in selected governmental hospitals and cardiac center Addis Ababa, Ethiopia 2021

Variables	Yes	No	AOR (95% CI)	*P*- value
**Wasting**				
**Age**				
0–12 months	40 (52.6%)	36 (47.4%)	1	
13 months-5 years	45 (32.4%)	94 (67.6%)	0.434 (0.231,0.816)	0.01*
6–15 years	59 (37.3%)	99 (62.7%)	0.684 (0.363,1.287)	0.239
**Sickness in the last two weeks**				
Yes	69 (53.1%)	61 (46.9%)	2.203 (1.367,3.551)	0.001*
No	75 (30.9%)	168 (69.1%)	1	
**Age of surgery**	–		1.072 (1.001,1.049)	0.046*
**Hgb level**	–		0.869 (0.792,0.955)	0.03*
**Stunting**				
**Fathers occupation**				
Employee	56 (33.7%)	110 (66.3%)	1	
Farmer	19 (61.3%)	12 (38.7%)	9.677 (0.945,99.094)	0.056
Merchant	10 (16.7%)	50 (83.3%)	0.384 (0.157,0.944)	0.037*
Self employed	22 (44.9%)	27 (55.1%)	1.810 (0.547,5.994)	0.332
**Bottle feeding**				
Yes	30 (34.1%)	58 (65.9%)	1	
No	22 (50%)	22 (50%)	2.993 (1.111,8.065)	0.03*
**Age of surgery**	- -		1.040 (1.007,1.074)	0.018*
**SPO**_**2**_ **level**			0.970 (0.943,0.998)	0.037*
**Underweight**				
**Age**				
0–12 months	46 (60.5%)	30 (39.5%)	1	
13 months-5 years	56 (46.3%)	83 (59.7%)	0.360 (0.183,0.711)	0.003*
6-10 years	41 (35%)	76 (65%)	0.439 (0.212,0.909)	0.027*
**Sickness in the last two weeks**				
Yes	63 (53.4%)	55 (46.6%)	1.834 (1.043,3.226)	0.035*
No	80 (37.4%)	134 (62.6%)		
**Pulmonary HTN**				
Yes	67 (53.2%)	59 (46.8%)	1.885 (1.094,3.246)	0.022*
No	76 (36.9%)	130 (63.1%)	1	
**Hgb**			0.814 (0.731,0.906)	0.00001*
**SPO**_**2**_	–		0.936 (0.906,0.968)	0.00008*

## Discussion

The purpose of this study was to assess the nutritional status of children with congenital heart disease in Addis Ababa’s designated hospitals. The prevalence of wasting and stunting was reported to be 144 (38.6%) and 134 (35.9%), respectively, and the prevalence of underweight in children under the age of 10 was 143(43.1%). Obesity and overweight were shown to be prevalent in 16 (4.3%) and 15 (4%), respectively, in this study.

The prevalence of underweight in the current study was 43.1 95% CI (37.72, 48.43%), which was similar to 42.5 and 44%, respectively, in studies conducted in Uganda and Egypt (22,37). The similarities could be due to the study environments being similar. In contrast, in a research conducted in London, the prevalence of underweight was reported to be 11% [4] among children who had undergone CHD surgery. The difference might be explained by the surgical intervention disparity, and the economic gap in the population could possibly have had a role. Furthermore, the prevalence in this study is higher than the 20.5% prevalence found in a Nigerian study (20). The difference could be due to the fact that children with acute and chronic illnesses other than CHD were excluded from the Nigerian study.

The prevalence of wasting in the present study is 144(38.6%) 95%CI (33.64, 43.57%). In consistent with this a study done in Egypt and Nigeria the prevalence was 37.5%,and 41.1% respectively [11, 15]. But in contrast to the present study a study in the Chile found only 12.1% [16]. This difference is might be explained by the subjects participated in the Chile have gotten treatment (surgery) early year of their life. The studies in Egypt also founds 6.7, and 23.8%of the participants to be wasted [17, 18] which had less prevalence compared to the present study the discrepancy is may be because a difference in clinical history of the patients as this study in Egypt excludes children with additional cardiac disorders and other disease. A study done in Hawassa Ethiopia reveals that 63% of the study subjects were wasted this is higher compared to the present study [12]. This variation might be because the study done on only un-operated and hospital admitted children it’s expected that children on OPD follow up to be healthier than who are admitted.

The prevalence of stunting in the present study is 134(35.9%) 95%CI (31.03, 40.82%). In contrast with this a study in Egypt found 61.9% of the participants to be stunted this shows a big discrepancy with the present study [18]. The difference of the study subjects may be the case for the gap that the study population in the study done in Egypt were un operated plus to this there was a difference in clinical characteristics like hemoglobin level the mean hemoglobin level was less than a result in this study which was mentioned to affect the nutritional status in different literatures. The results in the study done in Thailand shows less prevalence of stunting when compared with the current study which is about 16% [19] this might be because of the accessibility of treatment, distribution of CHD and economical difference among the population.

The prevalence of overweight and obesity in the present study reported to be 4 95%CI (2.2,6.3%) and 4.3% this was consistent with the result of the study done in Thailand in which the prevalence’s of overweight was 3% [19].

The commonest type of cardiac lesion from the acyanotic group of CHD in the present study was VSD which accounts for 36.7% and from the cyanotic CHD group TOF was the major type which accounts for 40%. Similar to this study done in India, Indonesia, Thailand, Iran, Egypt, Nigeria and Hawassa, Ethiopia which VSD and TOF was the major type of lesions from the acyanotic and cyanotic CHD respectively although there was a difference in prevalence VSD that ranges between (13–56%) and TOF (10.5–56%) [11, 12, 18–22]. The discrepancy may be explained by the difference in study setups most of the studies were single-center studies so the sample may be small in size to explain the real prevalence.

In the present study child age, being sick in the past 2 weeks, age at getting surgery, and hemoglobin level was associated with wasting. And stunting was found to be associated with occupation of the parent, bottle feeding, age at time of surgery, and level of SPO2. On the other hand underweight was associated with age, sickness, pulmonary hypertension, level of hemoglobin, and SPO2.

Compared to children aged 0–12 month children aged 13 months-5 years were less likely to be wasted in the present study [AOR = 0.434, 95% CI:(0.231,0.816)] likewise a study done in Hawassa Ethiopia also mentioned children under age 1 year are more likely to be malnourished [12]. also a study in China revealed that children age less than 1 years were more likely to be malnourished with high prevalence in the three indicators [23]. The similarity can be defined by that children under 1 years of age need more nutrients for growth and development than the older children and also the study setting were similar with the previous one.

Similar to the current study, studies done in India, Egypt, Nigeria and Ethiopia found a significant association between malnutrition and pulmonary hypertension, low SPO2 and low Hgb [11, 12, 15, 18, 20, 21, 24]. These reports are in line with our study the plausible explanation for the similarity is as oxygen and hemoglobin are useful in the metabolism of nutrients in a human body so decreasing of this two in the cells may cause disturbance in normal metabolism which may lead to malnutrition.

Compared to children who are not diagnosed with pulmonary hypertension children diagnosed with pulmonary hypertension were 1.885 times more likely to be underweight. This could be explained by that pulmonary hypertension increase energy requirement as the heart works hard to get blood to the lungs and the rest of the body against high pressure in the blood vessels and it also precipitates decreased nutritional intake and interrupts due to the feeling of fatigue and shortness of breath this could lead to malnourishment.

Age at surgery was associated with wasting and stunting in which children having treatment later have a relatively high risk of being malnourished [AOR = 1.072, 95%CI:(1.001,1.049)], and [AOR = 1.040, 95%CI:(1.007,1.074)] respectively. The results were consistent with the study in India, Thailand, and Nigeria older age at corrective surgery was one of the predictor of malnutrition. Different literatures reveals that early corrective surgery found to have a good impact on positive outcomes of nutritional status [19, 24, 25].

### Limitation

The sampling technique in this study was consecutive sampling technique. Using single 24 h recall method is also another limitation for this study because there will be misinformation or biased response.

## Conclusion

The prevalence of wasting, underweight and stunting among children with congenital heart disease in this study was found to be high. Child’s age, bottle feeding, being sick in the prior 2 weeks of the study, pulmonary hypertension, level of hemoglobin, level of SPO2 was the factors in this study which were found to be associated with malnutrition.

Hence the prevalence of wasting, stunting and underweight is high in children with CHD in the study settings. Therefore a need to action regarding to early intervention (surgery), giving a focused care for children under age of 12 months and children presented with other comorbidities. This will help decrease prevalence of malnutrition in children with CHD. Studying nutrient deficiency by using different types of dietary assessment techniques and including further laboratory investigations will help in getting a good result to know the severity.

## Data Availability

The data sets collected and analyzed for the current study are available from the corresponding author and can be obtained at a reasonable request.

## Abbreviations

ASD: Atrial Septal Defect
AVSD: Atrioventricular Septal Defect
BAZ: Body Mass for age Z score
CHD: Congenital Heart Defect/Disease
CoA: Coarctation of Aorta
HAZ: Height for Z-score
MUAC: Mid Upper Arm Circumference
PDA: Patent Ductus Arteriosus
PFO: Patent Foramen Ovale
PAH: Pulmonary Hypertension
PS: Pulmonary Stenosis
SAM: Sever Acute Malnutrition
SD: Standard Deviation
TAPVR: Total Anomalous Pulmonary Venous Return
TGA: Transposition of Great Artery
TOF: Tetralogy of Fallot
TR: Tricuspid Regurgitation
UNICEF: United Nations Children’s Fund
VSD: Ventricular Septal Defect
WAZ: Weight for Age Z-Score
WHZ: Weight for Height Z-Score
WHO: World Health Organization
